# Alanine aminotransferase as a predictor of adverse perinatal outcomes in women with intrahepatic cholestasis of pregnancy

**DOI:** 10.12669/pjms.322.9057

**Published:** 2016

**Authors:** Ali Ekiz, Basak Kaya, Muhittin Eftal Avci, Ibrahim Polat, Selin Dikmen, Gokhan Yildirim

**Affiliations:** 1 Ali Ekiz, Department of Maternal-Fetal Medicine Unit, Kanuni Sultan Suleyman Training and Research Hospital, Istanbul, Turkey; 2 Basak Kaya, Department of Maternal-Fetal Medicine Unit, Kanuni Sultan Suleyman Training and Research Hospital, Istanbul, Turkey; 3 Muhittin Eftal Avci, Department of Maternal-Fetal Medicine Unit, Kanuni Sultan Suleyman Training and Research Hospital, Istanbul, Turkey; 4 Ibrahim Polat, Department of Maternal-Fetal Medicine Unit, Kanuni Sultan Suleyman Training and Research Hospital, Istanbul, Turkey; 5 Selin Dikmen, Department of Obstetrics and Gynecology, Gokhan Yildirim, Kanuni Sultan Suleyman Training and Research Hospital, Istanbul, Turkey; 6 Department of Maternal-Fetal Medicine Unit, Kanuni Sultan Suleyman Training and Research Hospital, Istanbul, Turkey

**Keywords:** Intrahepatic cholestasis, Pregnancy, Adverse perinatal outcome, Alanine aminotransferase

## Abstract

**Objective:**

To evaluate the associations between adverse perinatal outcomes and serum transaminase levels at the time of diagnosis in patients with intrahepatic cholestasis of pregnancy.

**Methods:**

We performed a retrospective analysis of patients hospitalized for evaluation of intrahepatic cholestasis of pregnancy from January 2013 to June 2014 in a tertiary center. Seventy-one patients were divided into two groups according to the presence (Group I) or absence of adverse perinatal outcomes (Group II).

**Results:**

The mean aminotransferase levels and conjugated bilirubin levels at the time of diagnosis were significantly higher in Group I than in Group II. Receiver operating characteristic curve analysis revealed that the alanine aminotransferase level could predict adverse perinatal outcomes with 76.47% sensitivity and 78.38% specificity, and the cut-off value was 95 IU/L. Among patients with intrahepatic cholestasis of pregnancy, those with adverse perinatal outcomes were significantly older, had an earlier diagnosis, and had higher alanine aminotransferase levels. Using the 95-IU/L cut-off value, patients with intrahepatic cholestasis of pregnancy had a 3.54-fold increased risk for adverse perinatal outcomes.

**Conclusions:**

Patients with intrahepatic cholestasis of pregnancy and high alanineaminotransferase levels should be followed up for possible adverse perinatal outcomes.

## INTRODUCTION

Intrahepatic cholestasis of pregnancy (ICP) is the most common pregnancy-associated liver disease,^1^ and its incidence varies widely among various reports. In a study carried out in United Kingdom, the incidence of ICP was reportedly 0.7%.2ICP is characterized by pruritus, elevated serum aminotransferase levels, and/or elevated fasting serum bile acid levels. Pruritustypically occurs in the late second and third trimesters of pregnancy; itis worse at night and predominantly affects the palms and soles.^3^ Skin changes are limited to excoriations due to scratching. Jaundice is not a common finding in affected patients. These symptoms and biochemical abnormalities spontaneously resolve by postpartum week 4.^4^

The etiology and pathogenesis of ICP are not well defined. Genetic, hormonal, and environmental factors presumably play a role in the etiology of the disease.^5^ The disease usually occurs in genetically predisposed women who are exposed to elevated levels of steroid hormones and their metabolites during pregnancy.^3^ The characteristic feature of the disease is pruritus, and the diagnosis is verified by the demonstration of elevated liver aminotransferase levels or elevated fasting serum bile acid levels. Other causes of pruritus in pregnancy and abnormal liver function tests should be investigated and ruled out.^6^-^8^ There is no consensus about the most reliable biochemical test for diagnosing ICP.

ICP generally has a benign prognosis in pregnant women; nevertheless, it is associated with potential adverse perinatal outcomes, including spontaneous preterm delivery, meconium-stained amniotic fluid, nonreassuring fetal status, sudden fetal death, low APGAR scores, and neonatal respiratory distress syndrome independent of prematurity.^5^, ^8^-^10^ The aim in treating ICP is to relieve symptoms and prevent fetal morbidity and mortality. Ursodeoxycholic acid has been widely used for this purpose.^3^

In this study, we investigated the associations between adverse perinatal outcomes and serum transaminase levels at the time of diagnosis of ICP.

## METHODS

This retrospective study involved a thorough review of adverse perinatal outcomes in pregnant women with ICP who received obstetric care and delivered at Kanuni Sultan Suleyman Training and Research Hospital from January 2013 to June 2014. Patients with a diagnosis of ICP constituted the main population of this study and the ethics committee of our hospital approved the study.

The diagnosis of ICP was based on otherwise unexplained persistent pruritus without a rash in the late second or third trimester of pregnancy and abnormal liver function test results in keeping with the Royal College of Obstetricians and Gynaecologists guidelines for the diagnosis of ICP.^8^ A liver ultrasound examination was performed in all patients to exclude biliary obstructions. Other causes of liver disease, especially viral hepatitis, that may be associated with cholestasis and abnormal liver function test results were also investigated. Pregnancy-specific causes of elevated liver enzymes, such as preeclampsia and acute fatty liver of pregnancy, were excluded based on clinical and laboratory features.

### Exclusion criteria

multiple pregnancies, lack of follow-up, delivery in another hospital, incomplete medical records, and the presence of other diseases such as chronic liver diseases, ongoing viral infections affecting the liver, symptomatic cholelithiasis, skin diseases, and allergic disorders.

After establishment of the diagnosis, all patients were treated with ursodeoxycholic acid. Liver function tests were performed weekly. Fetal well-being was monitored by a modified biophysical profile weekly or biweekly;if necessary, Doppler ultrasonography was alsoper formed. Delivery was planned after 37 weeks of gestation.

Patient characteristics such as maternal age, gravidity, parity, gestational age at the time of diagnosis, delivery time, type of delivery, and history of ICP were extracted from the patients’ medical records. The biochemical parameters evaluated at the time of diagnosis were aspartate aminotransferase (AST), alanine aminotransferase (ALT), total bilirubin, unconjugated bilirubin, conjugated bilirubin, lactate dehydrogenase, hemoglobin, and hematocrit. The time between diagnosis and delivery and the period of biochemical recovery were also determined. When determining the transaminase normalization times, patients who delivered immediately after the diagnosis were excluded. Newborns’ APGAR scores, weight, and health status were obtained from the infants’ medical charts.

Adverse perinatal outcomes were defined as the presence of intrauterine fetal demise, preterm birth before 37 weeks of gestation, intrauterine growth restriction, fetal distress, meconium-stained amniotic fluid, neonatal intensive care unit admission, and placental abruption. The patients were divided into two groups based on the presence of at least one adverse perinatal outcome. Patients with at least one of these criteria were enrolled in Group I, and the others were enrolled in Group II.

The statistical analysis was carried out with MedCalc statistical software (version 13.3; MedCalc Software, Mariakerke, Belgium). Data are presented as mean±standard deviation. The Kolmogorov–Smirnov test was performed to assess the normality of the distribution of continuous variables. The chi-squared test and Fisher’s exact test were used to analyze categorical variables, and Student’s t-test was used to analyze normally distributed continuous variables. The Mann–Whitney U-test was used for non-normally distributed variables. Relative risk with the 95% confidence interval was also calculated. A p-value <0.05 was considered statistically significant. Receiver operating characteristic (ROC) curve was used to determine the cut-off value of ALT for predicting adverse perinatal outcomes.

## RESULTS

A total of 17,826 women gave birth at Kanuni Sultan Suleyman Training and Research Hospital from January 2013 to June 2014. During this 18-month period, the medical records of patients diagnosed with ICP were evaluated. In total, 164 patients were diagnosed with ICP. Two triple and eight twin pregnancies were excluded from the study. We enrolled 71 women with singleton pregnancies who were diagnosed with ICP and who met the inclusion criteria of the study. The incidence of ICP was 0.92%, with a mean incidence of 1 in 108 deliveries (164/17,826).

The pregnancy outcomes and clinical features of the 71 patients diagnosed with ICP were examined, and the patients were divided into two groups according to the presence or absence of adverse perinatal outcomes. Thirty-four patients with one or more adverse perinatal outcomes were enrolled in Group I, and 37 without adverse outcomes were enrolled in Group II.

The patients in Group I (29.44 years) were significantly older than those in Group II (26.38 years; p<0.05). There were no statistically significant differences in gravidity, parity, or body mass index between the two groups (Table-I). The mean hemoglobin (11.6 vs. 11.1 g/dl) and hematocrit levels (35.7% vs. 34.01%) were significantly higher in Group I than II (p<0.05).

**Table-I:** Clinical characteristics of the study groups.

Parameter (Mean ±SD)	Group 1 (n = 34)	Group 2 (n = 37)	*P* Value
Age	29.44±6.2	26.38±5.1	<0.05
Gravidity	2.53±1.4	2.22±1.4	0.24
Parity	1.12±1.4	0.73±0.9	0.44
GA at Diagnosis (week)	33.7±2.8	35.06±3.9	<0.05
GA at Delivery (week)	36.1±2.4	37.3±1.4	<0.05
DDI (day)	13.91±14.3	13.7±17	0.45
TN(day)	12.7±12.8	8.78±7.9	0.42
Birth Weight (g)	2835.88±626.7	3017.03±492.4	0.29
APGAR Score			
1st. Min.	7.94±1.1	8.27±0.9	0.23 0.37
5th. Min.	9.18±0.9	9.4±0.7	0.37

GA: Gestational Age, DDI: Diagnosis-Delivery Interval, TN: Transaminase Normalization.

The biochemical characteristics of the patients are shown in Table-II. The mean AST, ALT, and conjugated bilirubin levels at the time of diagnosis were significantly higher in Group I than in Group II (p< 0.01, p<0.01, and p<0.05, respectively). Patients in Group I were diagnosed with ICP significantly earlier than were those in Group II (33.70 vs. 35.06 weeks, respectively; p<0.05), and patients in Group I gave birth significantly earlier than did those in Group II (36.1 vs. 37.3 weeks, respectively; p<0.05).

**Table-II:** Biochemical characteristics of the study groups.

Parameter (Mean ±SD)	Group 1 (n = 34)	Group 2 (n = 37)	*P* Value
White Blood Cell(x10^3^/mm^3^)	9.77±2.69	10.62±2.92	0.208
Hemoglobin (g/dl)	11.67±1.16	11.07±1.24	<0.05
Hematocrit(%)	35.76±3.33	34.01±3.60	<0.05
Platelet Count (x10^3^/mm^3^)	236.51±82.11	255.71±81.57	0.327
AST (IU/L)	136.09±89.4	90.81±42.2	<0.01
ALT (IU/L)	213±175	114.4±93.2	<0.01
Total Bilirubin (µmol/L)	0.92±0.64	0.72±0.51	0.09
Conjugated Bilirubin (µmol/L)	0.62±0.5	0.41±0.3	<0.05

AST: Aspartate Aminotransferase, ALT: Alanineaminotransferase.

Six (8.4%) of the patients delivered before 34 weeks of gestation, and 19 (26.7%) delivered between 34 and 37 weeks of gestation. Intrauterine growth restriction occurred in two (2.80%) patients, and meconium-stained amniotic fluid was detected in five (7.04%) patients. One case of fetal demise and one case of placental abruption were observed among the patients in Group I. Six (8.4%) infants were admitted to the neonatal intensive care unit for transient tachypnea of the newborn and then discharged from the hospital uneventfully. The distributions of the adverse perinatal outcomes of the patients are shown in Table-III.

**Table-III:**
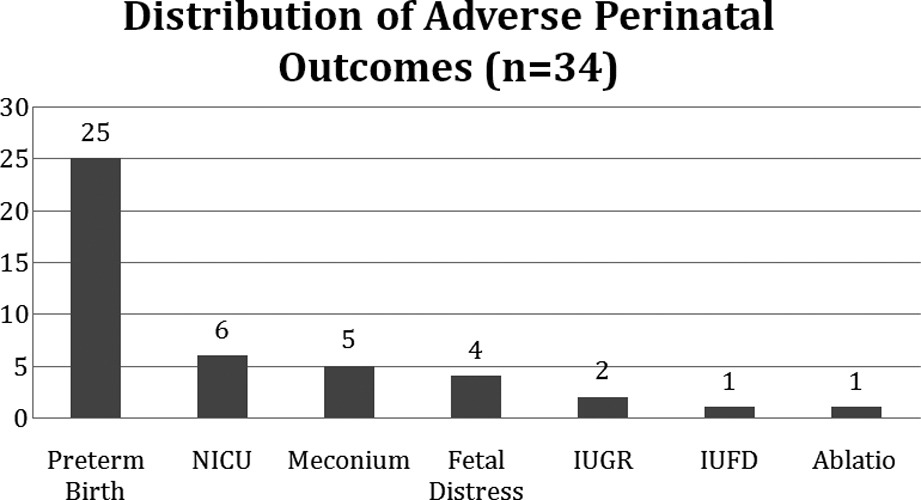
Distribution of adverse perinatal outcomes (n=34).

Maternal complications other than ICP included preeclampsia (7.04%, 5/71), HELLP syndrome (i.e., hemolysis, elevated liver enzyme levels, and a low platelet count; 1.40%, 1/71), and gestational diabetes mellitus (21.10%, 15/71). There was no significant difference in the history of ICP between the groups (p = 0.6). The incidence of preeclampsia and gestational diabetes was similar in both groups, and there were no significant differences in the history of preeclampsia, gestational diabetes, pregestational diabetes, or intrauterine fetal demise between the two groups.

**Fig.1:**
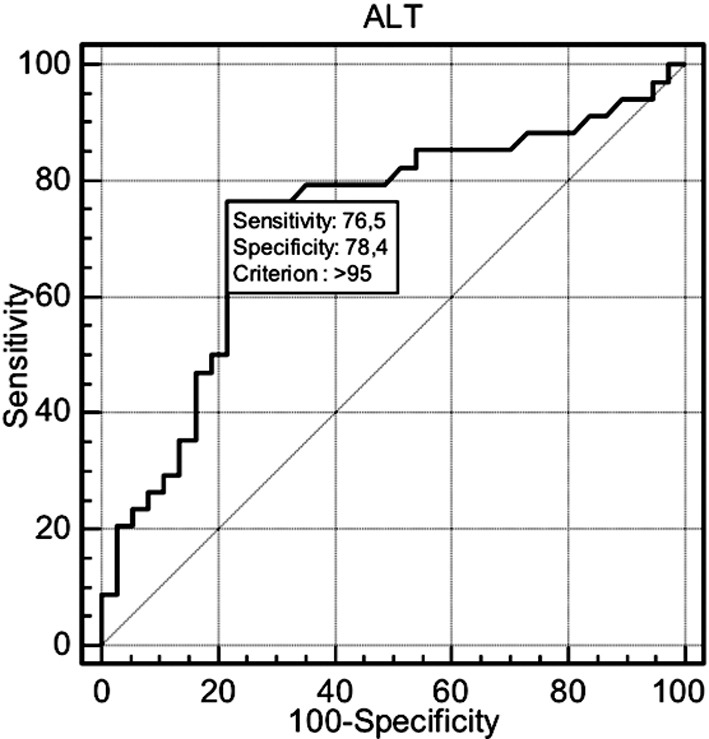
ROC Analysis.

When using ROC analysis, only ALT was found statistically significant with the level of >95 IU/L at the time of diagnosis of ICP and could be used as a predictor of adverse perinatal outcomes with a 76.47% sensitivity and 78.38% specificity (Fig.1). In addition, the positive likelihood ratio was calculated as 3.54.

## DISCUSSION

ICP, which is characterized by maternal pruritus in the absence of a rash and elevated serum aminotransferase levels or elevated fasting serum bile acid levels, is a liver disease specific to pregnancy.^3^ Geographic and ethnic variations are present in the incidence of ICP. In the present study, in accordance with the literature, the incidence of ICP was 0.92%.^2^ The etiology and pathogenesis of ICP are unknown, but are thought to be multifactorial.^5^

The associations between maternal and/or perinatal complications and ICP have been extensively studied. A recently published long-term population-based cohort study revealed that gestational diabetes and preeclampsia were strongly associated with ICP.^11^ In accordance with the literature; our study demonstrated an increased incidence of preeclampsia (8.4%) and gestational diabetes (21.1%) in association with ICP. In the same above-mentioned study,^11^ the authors found that women with ICP were generally of older age. In the present study, we also found that among women with ICP, those with adverse perinatal outcomes were significantly older hence elderly women with ICP may have a higher risk of developing adverse perinatal outcomes.

Many studies have reported associations between ICP and adverse fetal outcomes, including preterm delivery, a nonreassuring intrapartum fetal heart rate, meconium-stained amniotic fluid, and intrauterine fetal death.^7^, ^9^, ^12^, ^13^ In a recent study, a significant increase in the number of spontaneous and iatrogenic preterm deliveries was detected.^14^ The risk of spontaneous preterm delivery was slightly increased, and the majority of preterm deliveries were iatrogenic.^13^, ^15^ In the present study, preterm birth occurred before 34 weeks of gestation in 8.4% of patients and before 37 weeks in 26.7% of patients. In accordance with the literature, an increased rate of preterm delivery was detected in our study.

Although many studies have found that the risk of stillbirth is significantly higher in women than in those without ICP,^12^-^14^, ^16^ it has been suggested that active management of ICP by induction of labor before 38 weeks of gestation might decrease the incidence of stillbirth.^17^ In accordance with the literature, we observed only one stillbirth (1.4%); this patient already had gestational diabetes, and fetal demise was detected at 36 weeks of gestation. Similarly, Geenes et al.^14^ observed that seven of 10 women with stillbirth also had other pregnancy complications, and they concluded that the etiology of fetal death in these cases could be multifactorial. A suggested mechanism for sudden intrauterine fetal death is acute anoxia and fetal arrhythmia induced by elevated bile acids.^18^, ^19^

The underlying mechanisms associated with adverse perinatal outcomes in ICP are not well defined, but high levels of bile acids and their toxic metabolites are suspected to be associated with poor outcomes.^20^-^22^ Many studies have demonstrated a significant positive correlation between adverse fetal outcomes (including preterm delivery, meconium-stained amniotic fluid, and intrauterine fetal demise) and maternal fasting serum bile acid levels exceeding 40 mmol/L.^13^, ^14^, ^20^, ^23^

Other studies have shown a significant relationship of preterm delivery rates and fetal distress with levels of serum transaminases, especially ALT.^14^, ^24^, ^25^ Laatikainen et al.^25^ reported higher fetal distress rates with higher ALT levels. In a study that examined the clinical outcomes of ICP, the authors observed a positive correlation between preterm delivery rates and serum transaminase levels. A recent study by Geenes et al.^14^ also found a significant positive correlation between ALT levels and preterm delivery.

In this study, the AST, ALT, and conjugated bilirubin levels were significantly higher in patients as compared to those without adverse perinatal outcomes. We detected a significant positive correlation between ALT levels and adverse perinatal outcomes. Patients with ICP who had ALT levels>95 IU/L at the time of diagnosis had a 3.45-fold increased risk of adverse perinatal outcomes. This cut-off value may provide increased awareness of patients at increased risk of adverse perinatal outcomes.

In conclusion, elevated serum aminotransferase levels, especially elevated ALT levels, seem to be associated with adverse perinatal outcomes in patients with ICP, particularly those with preterm delivery. It should be considered that patients who are diagnosed with ICP in the earlier stages of gestation and who have elevated ALT levels >95 IU/L at the time of diagnosis have a higher risk of preterm delivery before 37 weeks of gestation and of other adverse perinatal outcomes described above.
